# Cardiac Hydatid Cyst: An Unusual Cause of Chest Pain

**Published:** 2013-12-01

**Authors:** Esref Tuncer, Ugur Turk, Emin Alioglu

**Affiliations:** 1 Department of Cardiology, Central Hospital, Izmir, Turkey

**Keywords:** Chest Pain, Cardiac Involvement

## Abstract

Hydatid disease is a parasitic infection caused by larvae of Echinococcus granulosus. Cardiac involvement in hydatid disease is uncommon, constituting only 0.5 - 2% of all cases of hydatidosis. Most patients with cardiac echinococcosis are asymptomatic, and the disease is often latent because a hydatid cyst in the heart grows very slowly. Only approximately 10 % of patients, especially those with large hydatid cysts, have clinical manifestations. Precordial pain is the one of the common symptoms and is most often vague and does not resemble angina pectoris.

## 1. Introduction

Hydatid disease is a parasitic infection caused by larvae of Echinococcus granulosus. Hydatid cysts can be located in various tissues, although they are most common in the liver and the lung. Cardiac involvement in hydatid disease is uncommon, constituting only 0.5 - 2% of all cases of hydatidosis. Areas of cardiac involvement in hydatid disease include the left ventricle (60% of cases), right ventricle (10%), pericardium (7%), pulmonary artery (6%), and left atrial appendage (6%); involvement of the interventricular septum is rare (4% of cases) (1). Right-sided cardiac hydatid cysts have characteristics different from those of left-sided cysts. Right-sided cysts have a tendency to expand intracavitarily and right ventricular cysts rupture more frequently, so they lead to pulmonary embolus, anaphylaxis, or sudden death. Rupture into the pericardial cavity can lead to pericarditis, effusion, and cardiac tamponade, whereas left-sided cysts tend to grow subepicardially. Most patients with cardiac echinococcosis have no symptoms, and the disease is often latent because a hydatid cyst in the heart grows very slowly. Unless a cyst is located in a critical anatomic site, the disease is usually diagnosed late (1). Signs and symptoms of cardiac hydatid cysts are extremely variable and directly related to the location and the size of the cysts. Only approximately 10% of patients, especially those with large hydatid cysts, have clinical manifestations. Precordial pain is the most common symptom and is most often vague and does not resemble angina pectoris.

## 2. Case Presentation

**Figure 1.**
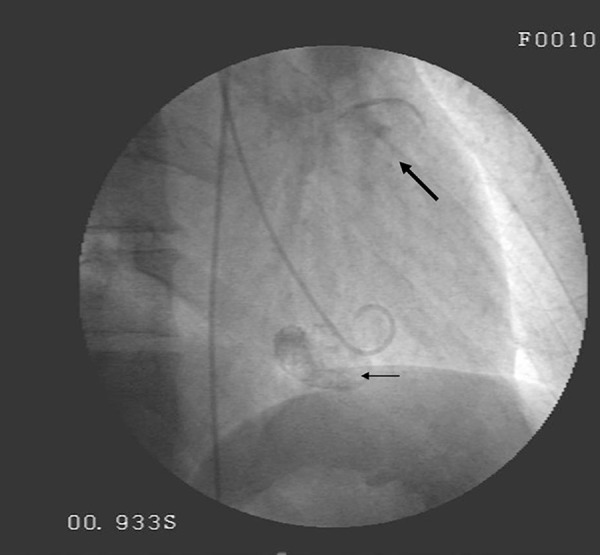
A Snapshot Image Which Demonstrating two Different Calcified Masses Before Left Ventriculography. (Arrows)

**Figure 2.**
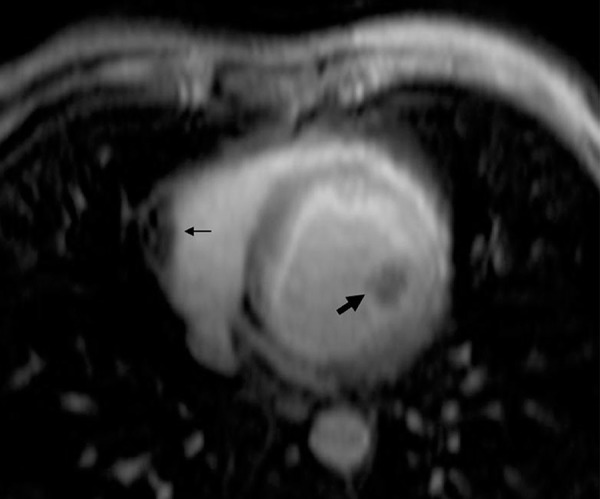
Cardiac Magnetic Resonance Imaging Showed Cystic Lesions, in the Left Ventricular Posterolateral Wall Protruding into the Lumen (Thick Arrow) and Right Ventricular Free Wall Expanding to Apical Segment. (Thin Arrow)

A 57-year-old man presented with a complaint of squeezing chest pain of 6 month-history. Physical examination was unremarkable. On transthoracic echocardiography, cystic mass was noted in the left ventricular wall. Diagnostic coronary angiography and fluoroscopy demonstrated normal epicardial coronary arteries and two different masses with calcified contours. (Figure 1) Serological test was negative for echinococcal disease. Cardiac magnetic resonance imaging showed cystic lesions, in the left ventricular posterolateral wall protruding into the lumen and right ventricular free wall expanding to apical segment (Figure 2). Abdominal ultrasonography and thorax CT revealed no any extracardiac cyst. The diagnosis of isolated cardiac hydatosis was made. After albendazole treatment for four weeks, surgery was performed for excision of the cyst. During the operation, rupture of the cyst was noted. The diagnosis of cardiac hydatid cyst was confirmed by pathological examination. During a six-month follow-up, the patient was asymptomatic, with no cystic appearance on transthoracic echocardiography.

## 3. Discussion

Cardiac hydatosis is associated with a high risk of potentially lethal complications (1, 2). Clinical manifestations and complications vary according to cyst location. Isolated cardiac cyst may be cured after surgery, while endovascular extracardiac involvement is associated with severe chronic complications (2). Cardiac hydatosis should be included in the differential diagnosis of cardiovascular disease in patients from endemic areas. Surgery remains the treatment of choice in the management of hydatid disease (3). Although antihelminthic drugs have been used in the preoperative and postoperative periods since 1977, extirpation of the lesion under cardiopulmonary bypass is recommended.
